# Studying the dynamics of the drug processing of pyrazinamide in *Mycobacterium tuberculosis*

**DOI:** 10.1371/journal.pone.0309352

**Published:** 2024-08-29

**Authors:** David Requena, Rydberg R. Supo-Escalante, Patricia Sheen, Mirko Zimic

**Affiliations:** 1 Laboratory of Bioinformatics and Molecular Biology, Laboratorios de Investigación y Desarrollo, Facultad de Ciencias e Ingeniería, Universidad Peruana Cayetano Heredia, Lima, San Martín de Porres, Peru; 2 Bioinformatics Group in Multi-Omics and Immunology, New York, NY, United States of America; 3 Department of Systems Biology, Columbia University, New York, NY, United States of America; Bennett University, INDIA

## Abstract

Pyrazinamide (PZA) is a key drug in the treatment of *Mycobacterium tuberculosis*. Although not completely understood yet, the bactericidal mechanism of PZA starts with its diffusion into the cell and subsequent conversion into pyrazinoic acid (POA) after the hydrolysis of ammonia group. This leads to the acidification cycle, which involves: (1) POA extrusion into the extracellular environment, (2) reentry of protonated POA, and (3) release of a proton into the cytoplasm, resulting in acidification of the cytoplasm and accumulation of intracellular POA. To better understand this process, we developed a system of coupled non-linear differential equations, which successfully recapitulates the kinetics of PZA/POA observed in *M*. *tuberculosis*. The parametric space was explored, assessing the impact of different PZA and pH concentrations and variations in the kinetic parameters, finding scenarios of PZA susceptibility and resistance. Furthermore, our predictions show that the acidification cycle alone is not enough to result in significant intracellular accumulation of POA in experimental time scales when compared to other neutral pH scenarios. Thus, revealing the need of novel hypotheses and experimental evidence to determine the missing mechanisms that may explain the pH-dependent intracellular accumulation of POA and their subsequent effects.

## Introduction

Tuberculosis (TB) is an infectious bacterial disease caused by *Mycobacterium tuberculosis* (MTB). According to the 2023 Global Tuberculosis Report by the World Health Organization, the annual global burden of TB comprises more than 10 million cases and 1.3 million deaths. Moreover, multidrug-resistant TB (MDR-TB) accounted for 410, 000 cases in 2022. Thus, TB represents one of the most critical public health challenges worldwide. TB transmission occurs from person to person via droplets expelled from the airways of individuals with active respiratory disease [1]. Following infection, some patients develop granuloma formation, a process in which MTB enters a dormant state and may persist for decades [2]. Nevertheless, infected individuals can develop active disease at any moment, process that is favored under conditions of weakened immunity [3].

Pyrazinamide (PZA) is a crucial drug utilized in the first-line treatment of drug-susceptible TB. Its main advantage lies in its ability to eliminate bacteria in a latent, non-growing state [4], exerting maximum bactericidal effects under acidic conditions [5–7]. Such conditions impact bacteria at sites of active inflammation [8, 9], or those residing within macrophages [10]. PZA is also used in treatment regimens of both MDR-TB [11] and extensively drug-resistant TB (XDR-TB) [12]. Therefore, the emergence of PZA-resistant isolates presents a significant public health concern. Consequently, it is imperative to comprehend its mechanism of action to counteract actual and potential adaptations of MTB leading to PZA resistance.

PZA is a pro-drug that enters the MTB cytoplasm via passive diffusion [13]. Intracellularly, the non-essential enzyme pyrazinamidase (PZase, encoded by the gene *pncA*) converts PZA into its acidic active form, pyrazinoic acid (POA) [14, 15]. Next, POA is expelled from the cell through an efflux pump system into the extracellular space. Proteins such as Rv0191, Rv3756c, Rv3008, and Rv1667c have been shown to participate in this efflux mechanism [16], among other associated proteins [17, 18]. Extracellularly, acidic conditions promote the protonation of a small fraction of POA, which subsequently reenters the bacteria and releases the proton into the cytoplasm [19]. This cycle of POA expulsion, protonation, and re-entry potentially results in the intracellular accumulation of POA and cytoplasmic acidification [19]. The combined effects of intracellular POA accumulation and cytoplasmic acidification are believed to disrupt the synthesis of vital cellular components and alter the central metabolism, the membrane permeability, causing energy failure and lethal consequences [13, 19, 20]. Nonetheless, although a high cytoplasmic acidification was expected to result from PZA treatment, it was demonstrated that mildly acidic pH conditions (between 5 and 6, known to induce PZA susceptibility) do not cause substantial cytoplasmic acidification [21]. The downstream molecular targets of POA have not been characterized yet, although several potential targets have been proposed, including the enzyme aspartate decarboxylase (PanD) [22], the fatty acid synthase I (FAS-I) [23, 24], and the ribosomal protein S1 (RpsA) [25]. Among them, the enzyme PanD, involved in the synthesis of coenzyme A (CoA) precursors [19, 26], is the candidate with most experimental support.

*In vitro*, PZA activity has demonstrated a dependence on an acidic extracellular environment with a pH below 6 [12, 27, 28]. Under neutral pH conditions, the sterilizing bacteriostatic effect is not observed [29]. Experiments providing radioactively labeled POA as a drug for MTB strain H37Ra found that an acidic environment effectively promotes POA accumulation [27]. *In vivo*, this acidic environment is facilitated within the macrophages where MTB resides during the inflammatory process, and may favor the protonation of extracellular POA contributing to recover and accumulate intracellular POA [30]. Additionally, it could aid in decreasing the membrane potential and reducing the bacterial metabolism, thus facilitating PZA activity [13]. Nonetheless, the acidic extracellular environment does not appear to be essential for the bactericidal effect of PZA, as overexpression of *pncA* [21], coincubation with the efflux pump inhibitors reserpine and valinomycin [31], and most recently constitutive activation of the cell envelope stress response [32] have shown to induce PZA susceptibility independent of environmental pH.

The primary cause of PZA resistance is the loss of PZase activity, resulting from critical mutations in *pncA* [14, 15, 33]. PZase is considered a non-essential enzyme that does not significantly impact the fitness of the organism [34, 35]. Mutations are dispersed throughout the entire sequence of *pncA*, although the most disruptive mutations affect the enzymatic active site or the metal binding site [12, 36]. PZase failure is also associated with mutations in its promoter, leading to a low expression level [28, 37]. Numerous efforts have been made to predict PZA resistance associated with PZase dysfunction using computational methods [38, 39]. However, the level of PZase activity is insufficient to fully explain the variability of PZA resistance [28, 40], suggesting alternative resistance mechanisms. It has been discovered that deviations in the POA efflux rate from a critical value towards higher values result in PZA resistance [41–44]. Similarly, intrinsic PZA resistance in *Mycobacterium smegmatis* is associated with a very active POA efflux mechanism, which has been found to be 900-fold higher than that of MTB strain H37Rv, preventing intracellular POA accumulation [27, 43, 44]. Moreover, resistance could be caused by mutations in downstream targets of POA, hindering proper interaction [19]. This has been observed for *rpsA* and *panD* genes in PZA-resistant strains of MTB with a wild-type (*wt*) *pncA* gene [22, 25]. However, the exact molecular mechanism by which these potential targets drive PZA susceptibility has not yet been established, but activation of the SigE-dependent cell envelope stress response has been suggested as the main driver of PZA susceptibility [32].

Mathematical modeling of molecular pathways and networks employing systems of non-linear, coupled differential equations serves as a valuable approach in systems biology research. It has been widely used for modeling complex metabolic pathways and concentration dynamics [45–47], drug kinetics and effectiveness [48]. In this study, we developed a mathematical model of the drug processing of PZA in MTB. This model examines the conversion of PZA into intracellular POA, the efflux-influx cycle of POA related to POA accumulation, and the potential cytoplasmic acidification. Overall, this study provides a better understanding of the quantitative impact of alterations in various steps of the processing of PZA, enabling the exploration of alternative modes of PZA resistance and susceptibility, and generating new hypotheses for subsequent investigations.

## Materials and methods

### Mathematical model of POA accumulation

**Fig 1:**
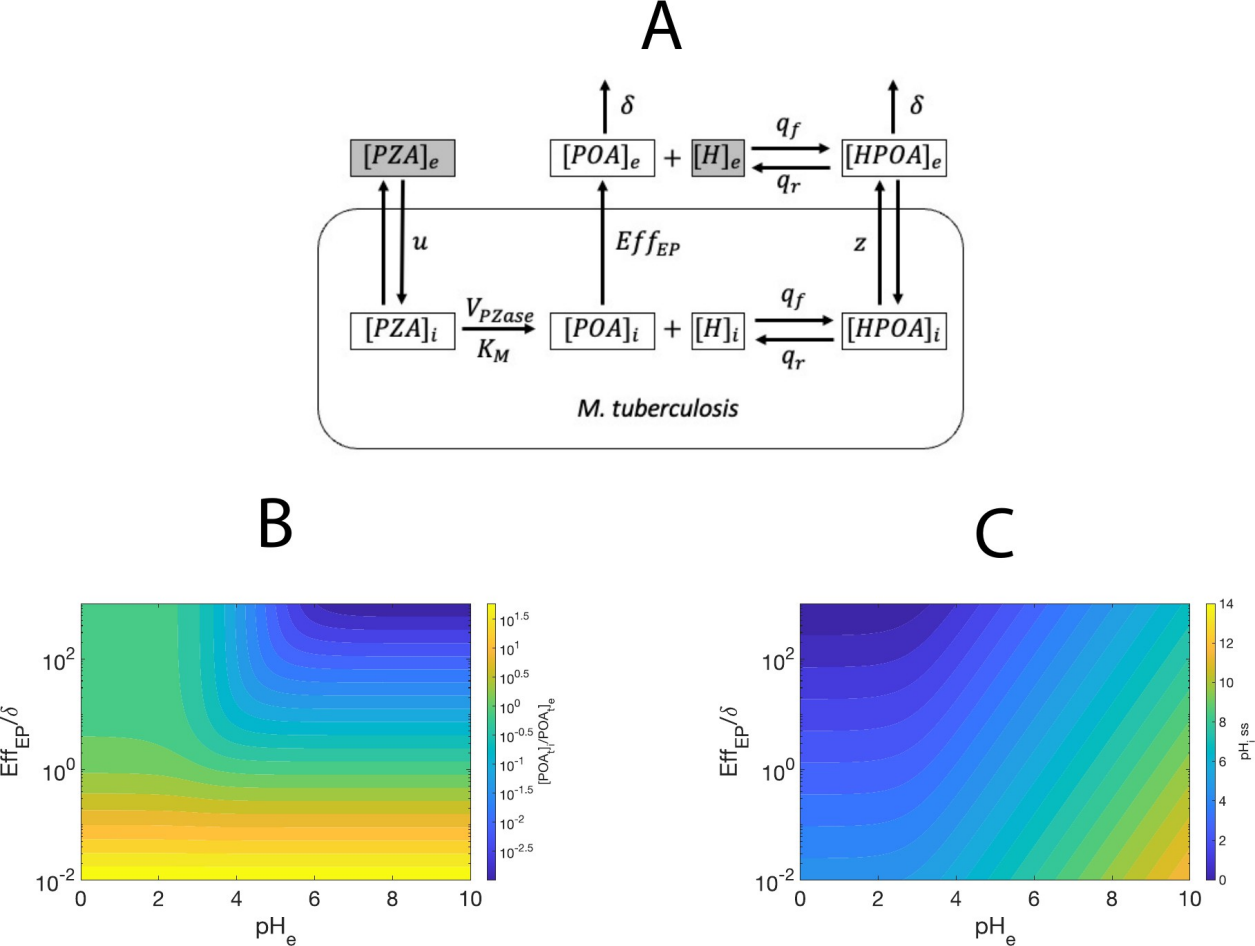
Mathematical model of PZA processing in *M*. *tuberculosis*. (A) Molecular pathway of the mechanism of accumulation and metabolism of pyrazinamide in *M*. *tuberculosis*. The arrows indicate the reactions and their directions. The letters next to the arrows represent the kinetic parameters involved in the reaction. The gray boxes indicate the molecular species that are assumed constant in the simulations and treated as fixed parameters. The [*POA*_*T*_]_*e*_ is depicted in their protonated (HPOA) and unprotonated (POA) states but treated as a single molecule in the system of equations. (B) Heatmap of the equilibrium relationships between the ratio [*POA*_*T*_]_*i*_*/*[*POA*_*T*_]_*e*_ and model parameters. (C) Heatmap of the equilibrium relationships between the *pH*_*i*_ and model parameters.

A mathematical model of the dynamics of PZA processing in MTB was built. The model considers the central elements of the currently accepted drug metabolism (Fig 1A): (1) the entry of extracellular PZA by passive diffusion, (2) the intracellular hydrolysis of PZA to unprotonated POA by the enzyme PZase, (3) the expulsion of intracellular unprotonated POA by an efflux pump system, (4) the loss of some extracellular POA, (5) the protonation of the extracellular unprotonated POA to form protonated POA (HPOA), and its subsequent diffusion into the cytosol, and (6) the protonation and deprotonation between intracellular POA and HPOA. We focused on the concentration of five molecular species, which are our state variables: 1) intracellular pyrazinamide ([*PZA*]_*i*_), 2) intracellular unprotonated POA ([*POA*]_*i*_), 3) intracellular protonated POA ([*HPOA*]_*i*_), 4) intracellular hydrogen ion ([*H*]_*i*_), and 5) the extracellular total POA ([*POA*_*T*_]_*e*_). This last variable is defined as the sum of extracellular protonated and unprotonated POA.

To assemble these variables into equations, we assumed:

1. The bacterium was assumed to be in a dormant state, neither growing nor replicating. So, the concentration of intracellular species is not reduced by dilution.
2. PZA is maintained at a steady level, reflecting a sustained-periodic intake during the anti-tuberculosis treatment or *in vitro* culture conditions, and enters by passive diffusion into the cytoplasm of MTB, represented by:
$$u({\left[PZA\right]}_{e}-{\left[PZA\right]}_{i})\;where\;u=diffusion\;constant$$ (1)
3. PZase activity is assumed independent of changes in the *pH*_*i*_. The intracellular enzymatic conversion of PZA into POA was modeled by Michaelis-Menten kinetics:
$$\frac{V_{PZAse}{\left[PZA\right]}_{i}}{K_{M}+{\left[PZA\right]}_{i}}$$ (2)
  Where *V*_*PZAse*_ is the maximum reaction rate, and *K*_*M*_ represents the Michaelis-Menten constant between PZase and PZA.
4. The POA efflux pump mechanism is assumed to never saturate at POA concentrations attainable inside the cell. Therefore, it operates at first order kinetics proportional to the concentration of intracellular unprotonated POA:
$${EP}_{eff}{\left[POA\right]}_{i}\;where\;{EP}_{eff}=efflux\;pump\;efficiency$$ (3)
5. The rate of the reversible protonation reaction between protonated and unprotonated POA was modeled using the law of mass action:
$$\left(q_{f}{\left[POA\right]}_{x}{\left[H\right]}_{x}-q_{r}{\left[HPOA\right]}_{x})\;where\;x=i(intracellular)\;or\;e(extracellular\right)$$ (4)
  Where *q*_*f*_, and *q*_*r*_ are the forward and reverse reaction rate constants, and their ratio is *K*_*a*_, the acid dissociation constant of POA. As protonation and deprotonation are known to be fast scale processes, large values for *q*_*r*_ and *q*_*f*_ were used.
6. Extracellular POA re-enters the mycobacterium only in its protonated from, justified by the fact that only uncharged molecules can traverse the cell membrane. Passive diffusion was assumed, modeling similarly to Eq (1)
$$z({\left[HPOA\right]}_{e}-{\left[HPOA\right]}_{i})\;where\;z=HPOA\;diffusion\;constant$$ (5)
  The rate at which protonated POA enters or exits the cell is considered proportional to the concentration difference between compartments.
7. The loss rate of total extracellular POA surrounding the bacterium is assumed linearly proportional to its concentration. This reflects *in vivo* conditions, because of the circulation system constantly diluting the local concentration of POA around the lung granuloma, where dormant mycobacteria are located. Also *in vitro* conditions, where the molecule migrates to areas with less concentration. Therefore:
$$\delta {\left[{POA}_{T}\right]}_{e}\;where\;\delta =clearing\;rate\;constant$$ (6)
8. The external pH is assumed constant, being supported by the homeostatic mechanisms of human hosts that regulate pH. Therefore, by the Henderson–Hasselbalch equation, [POA]e[HPOA]e is also constant. Setting Eq (4) to 0, [*POA*]_e_ can be expressed in terms of [*HPOA*]_e_. Then, [*POA*_*T*_]_e_ can be expressed as follows:
$${\left[{POA}_{T}\right]}_{e}={\left[HPOA\right]}_{e}+\frac{K_{a}{\left[HPOA\right]}_{e}}{{\left[H\right]}_{e}}$$ (7)
  Rearranging Eq (7):
$${\left[HPOA\right]}_{e}=\frac{{\left[H\right]}_{e}{\left[{POA}_{T}\right]}_{e}}{{\left[H\right]}_{e}+K_{a}}$$ (8)

Thus, a system of first-order non-linear differential equations was developed, modelling the concentration dynamics of each molecular specie.

### Non-linear optimization fitting with experimental data of cytoplasm acidification

The values of *K*_*M*_ (1240 *μM*) and *K*_*a*_(10^−2.9^ M) were retrieved from experimental data [29, 49]. The parameters u, z, *V*_*PZase*_,*Eff*_*EP*_, and *δ* were estimated using an experimental dataset of intracellular pH measurements in MTB after *in vitro* treatment with PZA [21]. Briefly, this dataset consists of two different experiments with triplicate treatments of 0, 80, 800, and 8000 *μ*M of PZA. The first experiment used *pH*_*e*_ = 5.8 and measured *pH*_*i*_ at 0, 8, 23, 46, 97, and 126 hours. The second experiment used a *pH*_*e*_ = 7 with a 100-fold overexpression of *pncA* and measured at 0, 7, 23, 47, 71, 95, and 123 hours [21]. To estimate the remaining kinetic parameters, a nonlinear curve-fitting was performed in Matlab R2023b using the lsqcurvefit function (Optimization Toolbox), minimizing the total sum of squares of the difference between experimental data and model predictions:

$$\underset{p}{\min}\sum_{i}{\left(F(p,t_{i})-y_{i}\right)}^{2}$$ (9)

Where *F*(*p*,*t*) corresponds to the model’s output at time *t*_*i*_ with parameter values provided in the vector *p*; and *y*_*i*_ corresponds to the experimental measurements at time *t*_*i*_.

The parameter intervals explored for *u*, *V*_*PZase*_, *z*, *Eff*_*EP*_, and *δ*, were [1*h*^−1^-10*h*^−1^], [10^2^*μMh*^−1^-10^4^*μMh*^−1^], [10^−3^*h*^−1^-10^−2^*h*^−1^], [10^3^*h*^−1^-10^4^*h*^−1^], and [10^−1^*h*^−1^-1*h*^−1^], respectively. The exploration was performed with a random seed of 2, which was used to choose the starting values from a log-uniform distribution on the provided bounds; performing a maximum of 800 function evaluations per iteration. The optimization problem was run until convergence.

### Numerical simulations, parameter sweep and equilibrium analysis

Simulations were carried out in Matlab, starting with an initial concentration of 0 for all the state variables, except for the *pH*_*i*_ which was set to 7.2 to reflect the average value in MTB under normal conditions. To understand the effect of different input values in the dynamics of the system, a bidimensional parameter sweep of [*PZA*]_*e*_ (1 nM up to 1000 M) and *pH*_*e*_ (4 to 8) was performed. The simulations were run for 240 and 10^7^ hours generating heatmaps of *pH*_*i*_ and [*POA*_*T*_]_*i*_ using the results of the bidimensional parameter sweep for both simulation times.

Equilibrium equations were calculated by setting all differential equations to 0 and solving for each state variable in the system. The equilibrium ratio between [*POA*_*T*_]_*i*_ and [*POA*_*T*_]_*e*_ was found to be a function of *pH*_*e*_ and *Eff*_*EP*_/*δ*. The equilibrium equations were used to quickly find the stationary concentration of each state variable in all the explored scenarios and to compare them with the simulation results.

### Exploration of scenarios of PZA resistance and susceptibility

To explore the system behavior in conditions associated with PZA resistance or susceptibility, parameters *V*_*PZase*_ and *Eff*_*EP*_ were modified to mimic experimentally observed alterations in the PZase enzyme and the POA efflux system, respectively. Resistance scenarios included reduction of PZase activity (a 100-fold reduction of the parameter *V*_*PZase*_) or increased efflux efficiency (a 100-fold increase of the parameter *Eff*_*EP*_). Susceptibility scenarios included increase in PZase activity (a 100-fold increase in the value of the parameter *V*_*PZase*_) or a reduction of efflux efficiency (a 100-fold decrease in the value of the parameter *Eff*_*EP*_). Simulations and equilibrium analysis was carried out as previously described for the *wt* scenarios.

## Results

### Mathematical model of POA accumulation

A mathematical model reflecting the concentration kinetics of the different molecular species involved in the processing of PZA in MTB was developed. It consists of a system of five first-order non-linear, coupled differential equations:

$$\frac{d{\left[PZA\right]}_{i}}{dt}=u({\left[PZA\right]}_{e}-{\left[PZA\right]}_{i})-\frac{V_{PZAse}{\left[PZA\right]}_{i}}{K_{M}+{\left[PZA\right]}_{i}}$$ (10)

$$\frac{d{\left[POA\right]}_{i}}{dt}=\frac{V_{PZAse}{\left[PZA\right]}_{i}}{K_{M}+{\left[PZA\right]}_{i}}-{EP}_{eff}{\left[POA\right]}_{i}-q_{f}{\left[POA\right]}_{i}{\left[H\right]}_{i}+q_{r}{\left[HPOA\right]}_{i}$$ (11)

$$\frac{d{\left[HPOA\right]}_{i}}{dt}=z(\frac{{\left[H\right]}_{e}{\left[{POA}_{T}\right]}_{e}}{{\left[H\right]}_{e}+K_{a}}-{\left[HPOA\right]}_{i})+q_{f}{\left[POA\right]}_{i}{\left[H\right]}_{i}-q_{r}{\left[HPOA\right]}_{i}$$ (12)

$$\frac{d{\left[H\right]}_{i}}{dt}=q_{r}{\left[HPOA\right]}_{i}-q_{f}{\left[POA\right]}_{i}{\left[H\right]}_{i}$$ (13)

$$\frac{d{\left[{POA}_{T}\right]}_{e}}{dt}={EP}_{eff}{\left[POA\right]}_{i}+z({\left[HPOA\right]}_{i}-\frac{{\left[H\right]}_{e}{\left[{POA}_{T}\right]}_{e}}{{\left[H\right]}_{e}+K_{a}})-\delta {\left[{POA}_{T}\right]}_{e}$$ (14)

This model simulates the main elements of PZA processing in MTB, including entry and hydrolysis of PZA by the enzyme PZase into POA, efflux-pump-mediated expulsion of POA, which is protonated and reenters as HPOA, and proton transfer between intracellular POA and HPOA (Fig 1A). The state variables and parameters are described in Tables 1 and 2, respectively.

**Table 1:** Symbol, description, and units of the molecules studied.

Symbol	Description	Units
[*PZA*]_*i*_	Intracellular concentration of pyrazinamide	*μM*
[*POA*]_*i*_	Intracellular concentration of unprotonated pyrazinoic acid	*μM*
[*HPOA*]_*i*_	Intracellular concentration of protonated pyrazinoic acid	*μM*
[*POA*_*T*_]_i_*	Intracellular concentration of total pyrazinoic acid: [*POA*]_*i*_+[*HPOA*]_*i*_	*μM*
[*POA*]_e_*	Extracellular concentration of unprotonated pyrazinoic acid	*μM*
[*HPOA*]_e_*	Extracellular concentration of protonated pyrazinoic acid	*μM*
[*POA*_*T*_]_*e*_	Extracellular concentration of total pyrazinoic acid: [*POA*]_*e*_+[*HPOA*]_*e*_	*μM*
[*H*]_*i*_	Intracellular concentration of hydrogen	*μM*

(*) These state variables were not explicitly considered in the model, but can be easily derived.

**Table 2:** Model parameters and fitted values.

Parameter	Meaning	Value	Units
*u*	Diffusion constant of PZA	1.0005	*h* ^−1^
*V*_*PZase*_	Maximum rate of intracellular POA production	838.7571	*μMh* ^−1^
*K*_*M*_	Michaelis-Menten constant between PZA and PZase	1240*	*μM*
*z*	Diffusion constant of HPOA	0.001	*h* ^−1^
*Eff*_*EP*_	Efflux pump efficiency of POA	2977.6	*h* ^−1^
δ	Clearing rate constant of extracellular POA	0.3162	*h* ^−1^
*K*_*a*_	Acid dissociation constant of POA: qrqf	10^−2.9^*	M
[*H*]_*e*_	Extracellular concentration of hydrogen	**	*μM*
[*PZA*]_e_	Extracellular concentration of pyrazinamide	**	*μM*

(*) Extracted from scientific literature.

(**) Dependent on the specific simulated scenario.

### Equilibrium analysis of the effect of pHeon[POAT]i[POAT]e

When the system reaches the equilibrium, the set of equations can be reduced to:

$$0=u∙{\left({\left[PZA\right]}_{i}\right)}^{2}+(V_{PZase}+u∙K_{M}-u∙{\left[PZA\right]}_{e})∙{\left[PZA\right]}_{i}-u∙K_{M}∙{\left[PZA\right]}_{e}$$ (15)

$$0=\frac{V_{PZase}∙{\left[PZA\right]}_{i}}{K_{M}+{\left[PZA\right]}_{i}}-{Eff}_{EP}∙{\left[POA\right]}_{i}$$ (16)

$$0=\frac{{\left[H\right]}_{e}∙{\left[{POA}_{T}\right]}_{e}}{{\left[H\right]}_{e}+K_{a}}-{\left[HPOA\right]}_{i}$$ (17)

$$0=q_{r}∙{\left[HPOA\right]}_{i}-q_{f}∙{\left[POA\right]}_{i}∙{\left[H\right]}_{i}$$ (18)

$$0={Eff}_{EP}∙{\left[POA\right]}_{i}-\delta ∙{\left[{POA}_{T}\right]}_{e}$$ (19)

The positive real root of Eq (15) is the equilibrium value of [*PAZ*]_i_. The flux of PZA to POA in the equilibrium was defined as:

$$V=\frac{V_{PZase}∙{\left[PZA\right]}_{i}}{K_{M}+{\left[PZA\right]}_{i}}$$ (20)

Which leads to a straightforward solution for the rest of equations:

$${\left[POA\right]}_{i}=\frac{V}{{Eff}_{EP}}$$ (21)

$${\left[H\right]}_{i}=\frac{{Eff}_{EP}}{\delta }∙(\frac{K_{a}∙{\left[H\right]}_{e}}{{\left[H\right]}_{e}+K_{a}})$$ (22)

$${\left[HPOA\right]}_{i}=\frac{V}{\delta }∙(\frac{{\left[H\right]}_{e}}{{\left[H\right]}_{e}+K_{a}})$$ (23)

$${\left[{POA}_{T}\right]}_{e}=\frac{V}{\delta }$$ (24)

Then, adding Eq (21) and Eq (22), and dividing by Eq (24), gives:

$$\frac{{\left[{POA}_{T}\right]}_{i}}{{\left[{POA}_{T}\right]}_{e}}=\frac{1}{\frac{{Eff}_{EP}}{\delta }}+\frac{{\left[H\right]}_{e}}{{\left[H\right]}_{e}+K_{a}}$$ (25)

In the right side of Eq (25), the first fraction corresponds to the intracellular unprotonated POA, while the second to the intracellular protonated POA recovered by re-entry through the cell wall. Whereas the first term could be unconstrainedly increased by reducing the efficiency of the efflux pump, the second term has an upper limit of 1 when [*H*]_e_ is much larger than *K*_*a*_. Additionally, the *pH*_*e*_ shows a significant effect on Eq (25) only when *Eff*_*EP*_/*δ* is greater than 1 (Fig 1B; S1A Fig). Solving Eq (25) for [*POA*_*T*_]_*i*_:

$${\left[{POA}_{T}\right]}_{i}=\frac{V}{\delta }(\frac{1}{\frac{{Eff}_{EP}}{\delta }}+\frac{{\left[H\right]}_{e}}{{\left[H\right]}_{e}+K_{a}})$$ (26)

There, [*POA*_*T*_]_*i*_ could be increased by raising *V*, the flux of PZA to POA, which can be achieved by overexpression of PZase or mutations that increase the catalytic activity of PZase. Overall, this equation provides a reasonable approximation which allows to understand the effect of different experimental parameters on [*POA*_*T*_]_*i*_in MTB.

### Parameter sweep of [*PZA*]_*e*_ and *pH*_*e*_ in *wt* MTB indicates a pH-dependent POA accumulation attainable at equilibrium but not at experimental timescales

**Fig 2:**
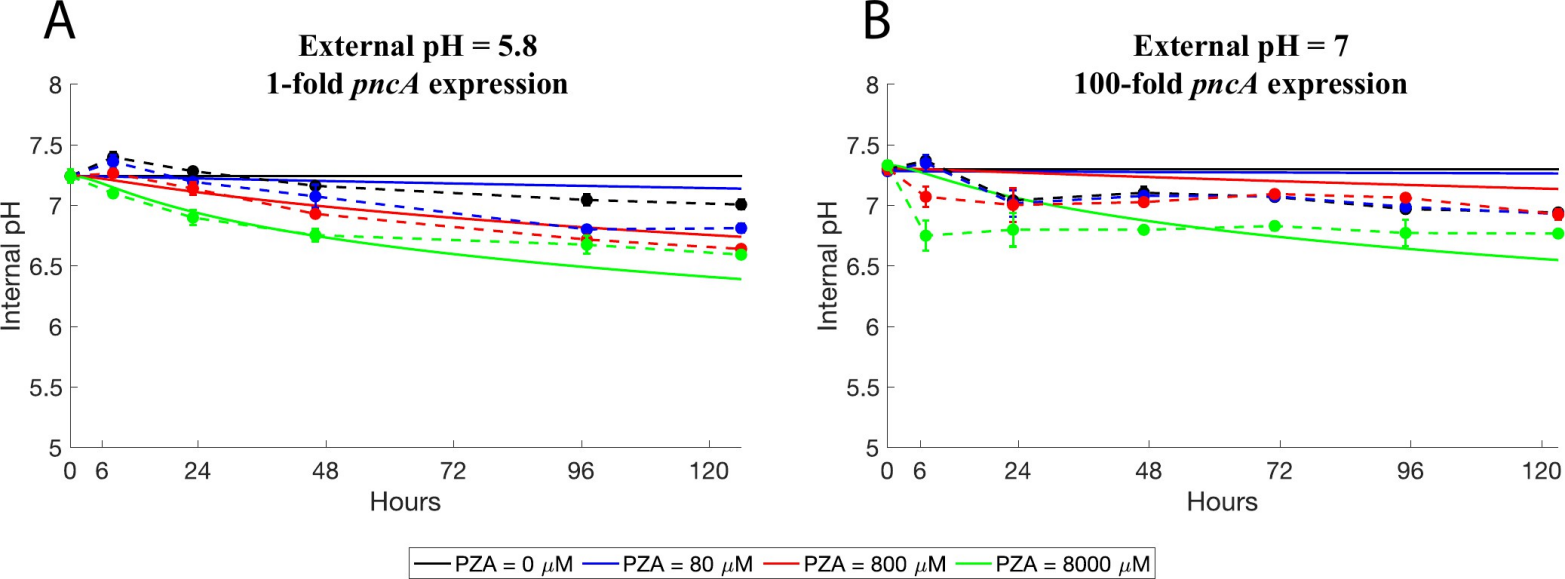
Parameter estimation using cytoplasmic acidification data. Experimental and fitted curves of different PZA treatments, under (A) *pH*_*e*_ = 5.8, and (B) *pH*_*e*_ = 7 and a 100-fold expression of the gene *pncA*.

**Fig 3:**
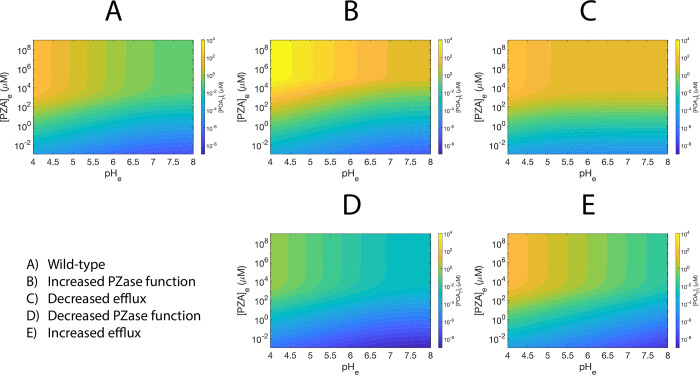
Heatmaps of the predicted [*POA*_*T*_]_*i*_ at equilibrium for the five scenarios studied. (A) Wild-type scenario. (B) Increased PZase activity. (C) Decreased efflux. (D) Decreased PZase activity. (E) Increased efflux. The color scale is the same in all panels.

To start exploring the dynamics of the model, parameters were fitted using available data on cytoplasmic acidification. The parameter values obtained are displayed in Table 2, and the fitting least square error was 5.99 (Fig 2A and 2B). After that, the effect of different values of [*PZA*]_*e*_ and *pH*_*e*_ on [*POA*_*T*_]_*i*_ at equilibrium were explored, using conditions reflecting MTB strain H37Ra (Fig 3). The highest concentration reached by [*POA*_*T*_]_*i*_ in the explored parameter space is ~200 μM and it is achieved for low extracellular pH and high concentrations of [*PZA*]_*e*_ (Fig 3A). This parameter sweep demonstrated how, in contrast to neutral conditions, acidic conditions give the system the ability to achieve higher [*POA*_*T*_]_*i*_ for similar [*PZA*]_*e*_ values. This becomes more evident for higher values of [*PZA*]_*e*_, at which the PZase enzyme is expected to be saturated by its substrate. Additionally, the cytoplasmic acidification at equilibrium only depended on *pH*_*e*_ (S2A Fig), and a high acidification is predicted even for neutral environments, with almost all *pH*_*i*_ at equilibrium being lower than 4.

**Fig 4:**
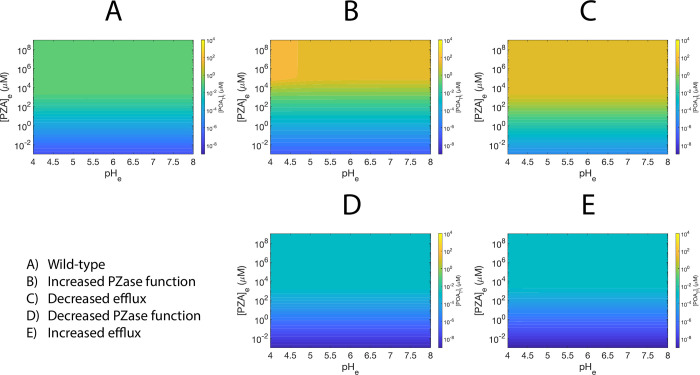
Heatmaps of predicted [*POA*_*T*_]_*i*_ at 240 hours of simulation, for the five studied scenarios. (A) Wild-type scenario. (B) Increased PZase activity. (C) Decreased efflux. (D) Decreased PZase activity. (E) Increased efflux. The color scale is the same in all panels.

**Fig 5:**
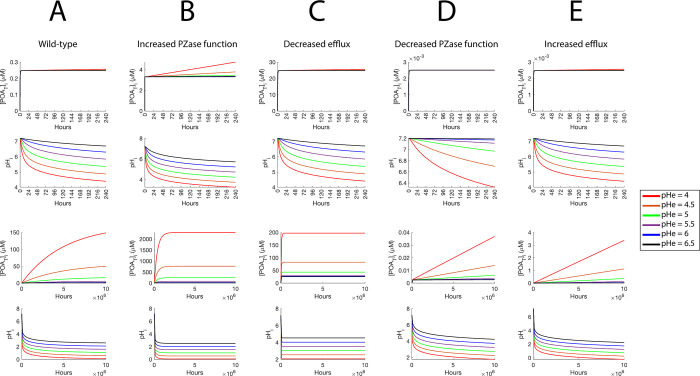
Concentration trajectories for the five explored scenarios at different time scales. (A) Wild-type scenario. (B-C) Simulated PZA-susceptibility scenarios: (B) increased PZase activity, and (C) reduction of efflux efficiency. (D-E) Simulated PZA-resistance scenarios: (D) reduction of PZase activity, and (E) increased efflux efficiency. In each column, the first and second plot correspond to the short-term dynamics (240 hours) of [*POA*_*T*_]_*i*_ and *pH*_*i*_, respectively. Analogously, the third and fourth plot correspond to the long-term dynamics (10^7^ hours).

Contrarily, after 240 hours of simulation, the *pH*_*i*_ obtained is between 4 and 7 for most of the explored parameter space, showing moderate acidification, thus matching experimental evidence (S3A Fig). Interestingly, the heatmaps of [*POA*_*T*_]_*i*_ indicate that equilibrium is far from being reached, and that [*POA*_*T*_]_*i*_ behaves independently of *pH*_*e*_ (Fig 4A). Similarly, simulation trajectories at 240 hours illustrate that *pH*_*e*_ values representing strong acidic conditions do not help to accumulate more [*POA*_*T*_]_*i*_ than *pH*_*e*_ values in neutral conditions, although slight intracellular acidification is observed (Fig 5A). When simulations run for an extremely long period of time (10^7^ hours), the effect of *pH*_*e*_ becomes visible and the cytoplasmic acidification becomes stronger. However, the time needed to reach this state is experimentally unrealistic (Fig 5A).

### Resistance and susceptibility scenarios display different regimes of accumulation of [*POA*_*T*_]_*i*_ and cytoplasmic acidification

The effect of *pH*_*e*_ in increasing [*POA*_*T*_]_*i*_ is present with different strength in all the evaluated susceptibility and resistance scenarios in the equilibrium at saturating [*PZA*]_*e*_ (Fig 3A–3E). In the equilibrium, the susceptibility scenarios (a 100-fold increase in PZase activity or 100-fold reduction of efflux efficiency), show that the values of [*POA*_*T*_]_*i*_ obtained at neutral conditions are similar to those in highly acidic conditions in the *wt* scenario (Fig 3B, 3C). Additionally, in the scenario of reduced efflux efficiency, [*POA*_*T*_]_*i*_ displayed less dependence on *pH*_*e*_ (Fig 3C; S4C Fig) in the explored range. Conversely, the scenario of increased PZase activity showed a greater dependence on *pH*_*e*_ to reach the maximum [*POA*_*T*_]_*i*_ possible (Fig 3B; S4B Fig), which is predicted to be higher than in the scenario of reduced efflux efficiency (S4B, S4C Fig). Also, in the equilibrium, the resistance scenarios (a 100-fold reduction of PZase activity or a 100-fold increase in efflux efficiency) show different behaviors. For the scenario of reduced PZase activity, even at the most acidic conditions explored (pH = 4), the maximum [*POA*_*T*_]_*i*_ is similar to the values obtained at neutral conditions in *wt* (Fig 3D; S4D Fig). However, for the scenario of increased efflux efficiency, the same range of values of [*POA*_*T*_]_*i*_ in the *wt* scenario could be achieved in acidic conditions, but not at neutral conditions (Fig 3E; S4E Fig). Cytoplasmic acidification at equilibrium is never affected by variations in PZase activity and is independent of [*PZA*]_*e*_ (S2B and S2D; S5B and S5D Figs), but is lower when the efflux efficiency is reduced (S2C; S5C Figs), and higher when the efflux efficiency is increased (S2E; S5E Figs).

Noticeably, the effect of *pH*_*e*_ in [*POA*_*T*_]_*i*_ is mostly absent in all the simulations at 240 hours (Fig 4), being barely present in the susceptibility scenario of increased PZase activity (Fig 4B; S6B Fig). In this short simulation time scale, both susceptibility scenarios already have higher [*POA*_*T*_]_*i*_ than in the *wt* scenario (Fig 4B, 4C; S6B, S6C Fig). The heatmaps of both resistance scenarios are similar, displaying lower values of [*POA*_*T*_]_*i*_ than the *wt* scenario in the explored parameter range (Fig 4D, 4E; S6D, S6E Fig). Also, at 240 hours, variations in the efflux efficiency result in similar cytoplasmic acidification to the *wt* scenario (S3C and S3E; S7C and S7E Figs), but null acidification is observed in the scenario of reduced PZase activity, even at acidic conditions (S3D; S7D Figs). Conversely, in the scenario of increased PZase activity, higher acidification than in the *wt* scenario could be obtained even at neutral *pH*_*e*_ for high values of [*PZA*]_*e*_ (S3B; S7B Figs).

**Fig 6:**
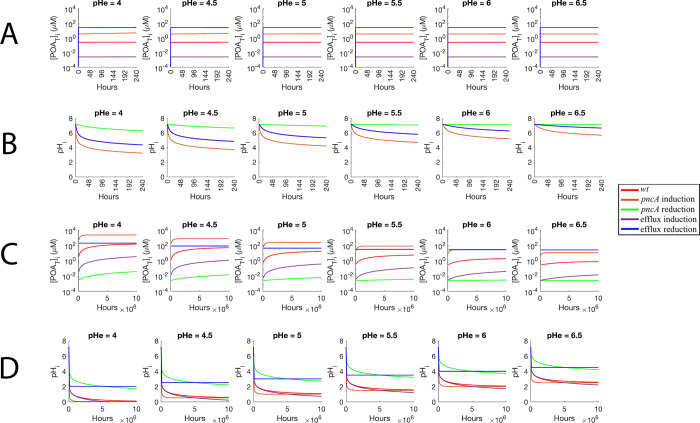
Simulated trajectories of the five explored scenarios at different values of *pH*_*e*_. Short-term dynamics (240 hours) of (A) [*POA*_*T*_]_*i*_, and (B) *pH*_*i*_. Long-term dynamics (10^7^ hours) of (C) [*POA*_*T*_]_*i*_, and (D) *pH*_*i*_.

Finally, the [*POA*_*T*_]_*i*_ and *pH*_*i*_ of the susceptibility, resistance, and *wt* scenarios were compared at different *pH*_*e*_ values (Figs 5; 6). The resistance scenarios have the same dynamics of [*POA*_*T*_]_*i*_ at 240 hours, independently of the *pH*_*e*_ value (Figs 5D, 5E; 6A). At 10^7^ hours, the resistance scenario of reduced PZase activity has much lower [*POA*_*T*_]_*i*_ than the scenario of increased efflux efficiency, but none of them reached equilibrium and are still orders of magnitude below the concentrations reached by the *wt* (Figs 5D, 5E; 6C). On the other hand, at 240 hours, both susceptibility scenarios have higher [*POA*_*T*_]_*i*_ than the *wt*, with the scenario of increased PZase activity displaying lower [*POA*_*T*_]_*i*_ than the scenario of reduced efflux efficiency (Figs 5B, 5C; 6A). However, the opposite is seen at 10^7^ hours, although the scenario of reduced efflux efficiency equilibrates faster (Figs 5B, 5C; 6C). As the *pH*_*e*_ is reduced, the *wt* scenario approaches the susceptibility scenario of reduced efflux efficiency, suggesting that acidic conditions make the *wt* behave like this scenario (Fig 6C). For cytoplasmic acidification, at 240 hours the *wt* and scenarios with variations in efflux have similar acidifications levels (Figs 5A and 5C–5E; 6B). Almost null acidification is observed for the scenario of reduced PZase activity, while the scenario of increased PZase activity causes the highest acidification (Figs 5B and 5D; 6B). At 10^7^ hours, acidification is shown to be strong for all simulated scenarios, with the scenarios of reduced PZase activity and reduced efflux efficiency being the most conservative scenarios (Figs 5C, 5D; 6D), and the scenario of increased efflux efficiency leading to the highest acidification (Figs 5E; 6D).

## Discussion

In this study, we are presenting a mathematical model reflecting the known elements of the metabolism of PZA in MTB. Model parameters were fitted to experimental data, successfully recapitulating the intracellular acidification observed in wild-type MTB. Using this model, the impact of having an acidic extracellular environment on the dynamics of POA concentration and cytoplasmic acidification was quantitively assessed. Subsequently, the model dynamics was explored under different scenarios of extracellular PZA concentration and extracellular pH, studying conditions leading to PZA resistance. There are two potential roles for the acidic extracellular environment in PZA susceptibility: 1) cytoplasmic acidification, which has been suggested to be non-significant [21], and 2) pH-dependent accumulation of intracellular POA [27]. We investigated these effects in our model, which predicts that at equilibrium, when *pH*_*e*_ is below 7, [*POA*_*T*_]_*i*_ increases with the extracellular acidity. However, when *pH*_*e*_ is 7 or above, it solely depends on [*PZA*]_*e*_, and the contribution of *pH*_*e*_ becomes negligible. This agrees with previous experimental results. Zhang *et al*. showed that when POA is provided in the extracellular medium, an acidic pH favors the intracellular accumulation of C14-radiolabelled POA [27]. However, they did not distinguish between the protonated and unprotonated forms of POA. Our model provides further insight, showing that at equilibrium *pH*_*e*_ only contributes to accumulate the protonated form. And, by the Henderson-Hasselbalch equation, this would lead to high intracellular acidification with low pH. Conversely, only slight cytoplasmic acidification has been observed in *wt* MTB, even at acidic environments for 120 hours [21]. In line with this, neither high cytoplasmic acidification nor dependence between [*POA*_*T*_]_*i*_ and *pH*_*e*_ in the first 10 simulated days were observed. Overall, this suggests that an alternative POA accumulation mechanism without intracellular acidification should be in place. Efflux reduction caused by growth arrest and reduced metabolic activity after culturing *M*. *tuberculosis* in a low pH medium is a potential explanation. This would agree with the general observation that susceptibility is mainly achieved under conditions that induce stress and reduce the metabolic activity of *M*. *tuberculosis* [32]. If a mechanism for the accumulation of intracellular unprotonated POA (dependent on an acidic environment) exists, then the equilibrium could be achieved within the first 10 days of simulation, without changing the results for all simulated scenarios.

Additionally, scenarios associated with increased PZA susceptibility (mutants with reduced efflux efficiency or increased PZase activity) and with PZA resistance (mutants with increased efflux efficiency or reduced PZase activity) were explored. Under the same conditions, the mutants associated with increased PZA susceptibility achieved higher concentrations of intracellular *POA*_*T*_ than *wt* MTB, while mutants associated with PZA resistance achieved lower concentrations. The scenarios of PZA resistance explored have been observed in nature: 1) PZA resistance in *M*. *tuberculosis* is predominantly associated with a reduction in PZase activity due to missense mutations in the gene *pncA* and, less commonly, mutations in the promoter [28, 40, 50, 51]. And 2) PZA resistance due to an elevated efflux activity has been observed in *M*. *smegmatis*, which exhibits a POA efflux rate approximately 900-fold higher than that of the *M*. *tuberculosis* strain H37Rv [43]. Similarly, increased PZA susceptibility has been achieved *in vitro* by 1) the use of efflux inhibitors like reserpine, valinomycin or piperine [27, 32]; and 2) overexpression of *pncA* [52]. Additionally, our simulations showed that alterations in efflux efficiency do not influence cytoplasmic acidification in usual experimentation time scales (less than 240 hours). However, higher PZase activity expedites acidification, while lower PZase activity significantly decelerates the process.

Our model, and the conclusions made from it, are limited by the data available and the assumptions made. Steps lacking evidence were not considered, like that the efflux pump is thought to expel only unprotonated POA, but not protonated POA, which is an uncontested assumption in the field. Considering that, intracellularly, the majority of POA exists in an unprotonated state, most POA molecules expelled by the efflux pump would be unprotonated. However, its ability to also expel protonated POA still needs to be resolved. Although our assumptions are sound under the current knowledge, they may differ from reality as new evidence arises or more complexity is considered. However, the main conclusions of our study could be regarded as an upper bound of the system’s ability to increase [*POA*_*T*_]_*i*_.

Finally, while it is crucial to accurately determine the concentrations of the molecular species involved in the processing of PZA to assess MTB susceptibility, quantifying these molecules remains as a complex task, even *in vitro*. The mathematical model presented herein serves as a valuable tool to quantitatively study the effect of different alterations in the molecular processing of PZA, or different conditions of *pH*_*e*_ and [*PZA*]_*e*_ in [*POA*_*T*_]_*i*_. This model proves beneficial not only for quantitively estimating PZA susceptibility and resistance in MTB as response to diverse biological scenarios but also for comprehending the dynamics of the biochemical reactions underlying the antibiotic action of PZA.

## Conclusions

We have developed the first mathematical model of the molecular processing of PZA in *M*. *tuberculosis*, using a system of ordinary differential equations. This model demonstrated that, although an acidic environment has the potential to promote accumulation of protonated POA and cytoplasmic acidification in MTB, this will not happen at regular experimental time scales. Our findings explain the lack of substantial cytoplasmic acidification after PZA treatment in MTB observed experimentally. Thus, revealing the need of an alternative mechanism to explain the pH-dependent accumulation of intracellular POA. Future research should study the dependence of the efflux efficiency on the extracellular pH. Additionally, the model has proven advantageous for quantitatively assessing the impact of alterations in the molecular processing of PZA, simulating scenarios of susceptibility and resistance. Hence, serving as a tool to generate and simulate novel hypotheses.

## Supporting information

S1 Fig Relationship between model parameters at equilibrium. (A) The ratio [*POA*_*T*_]_*i*_/[*POA*_*T*_]_*e*_ at equilibrium as a function of *pH*_*e*_ and the ratio *Eff*_*EP*_/*δ*. (B) Values of *pH*_*i*_ at equilibrium, as a function of *pH*_*e*_ and the ratio *Eff*_*EP*_/*δ*.
(TIF)

S2 Fig Heatmaps of the predicted *pH*_*i*_ at equilibrium for the five studied scenarios. (A) Wild-type MTB. (B) Increased PZase activity. (C) Decreased efflux. (D) Decreased PZase activity. (E) Increased efflux. The color scale is the same in these five panels.
(TIFF)

S3 Fig Heatmaps of the predicted *pH*_*i*_ at 240 hours of simulation for the five studied scenarios. (A) Wild-type MTB. (B) Increased PZase activity. (C) Decreased efflux. (D) Decreased PZase activity. (E) Increased efflux. The color scale is the same in these five panels.
(TIFF)

S4 Fig Heatmaps of the predicted [*POA*_*T*_]_*i*_ at equilibrium for the susceptibility/resistance studied scenarios. (A) Wild-type MTB. (B) Increased PZase activity. (C) Decreased efflux. (D) Decreased PZase activity. (E) Increased efflux.
(TIFF)

S5 Fig Heatmaps of the predicted *pH*_*i*_ at equilibrium for the susceptibility and resistance studied scenarios. (A) Wild-type MTB. (B) Increased PZase activity. (C) Decreased efflux. (D) Decreased PZase activity. (E) Increased efflux.
(TIFF)

S6 Fig Heatmaps of the predicted [*POA*_*T*_]_*i*_ at 240 hours of simulation for the susceptibility and resistance studied scenarios. (A) Wild-type MTB. (B) Increased PZase activity. (C) Decreased efflux. (D) Decreased PZase activity. (E) Increased efflux.
(TIFF)

S7 Fig Heatmaps of the predicted *pH*_*i*_ at 240 hours of simulation for the susceptibility and resistance studied scenarios. (A) Wild-type MTB. (B) Increased PZase activity. (C) Decreased efflux. (D) Decreased PZase activity. (E) Increased efflux.
(TIFF)
