# Supplementary figures and images for: Studying the dynamics of the drug processing of pyrazinamide in Mycobacterium tuberculosis
Source: PLoS One. 2024 Aug 29;19(8):e0309352. doi: 10.1371/journal.pone.0309352 (PMC11361689; doi:10.1371/journal.pone.0309352)

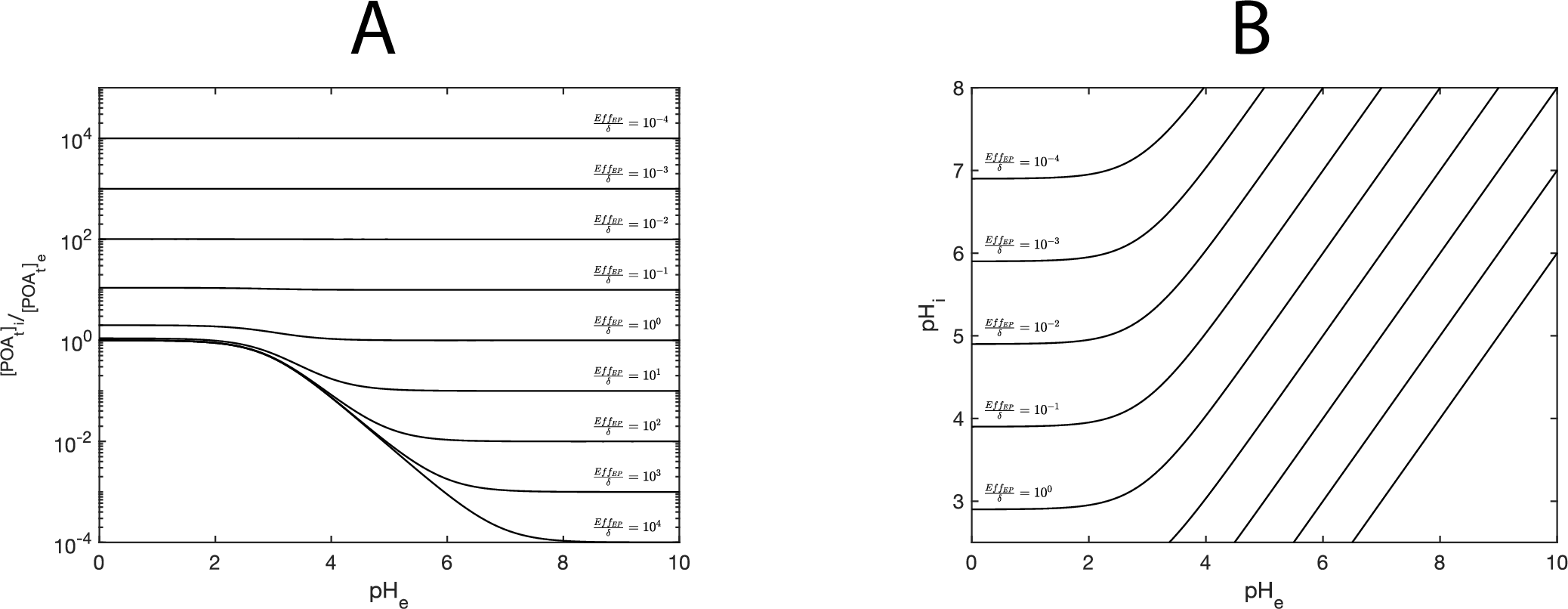

Supplement: S1 Fig — (A) The ratio [POAT]i/[POAT]e at equilibrium as a function of pHe and the ratio EffEP/δ. (B) Values of pHi at equilibrium, as a function of pHe and the ratio EffEP/δ. (TIF) [file pone.0309352.s001.tif]

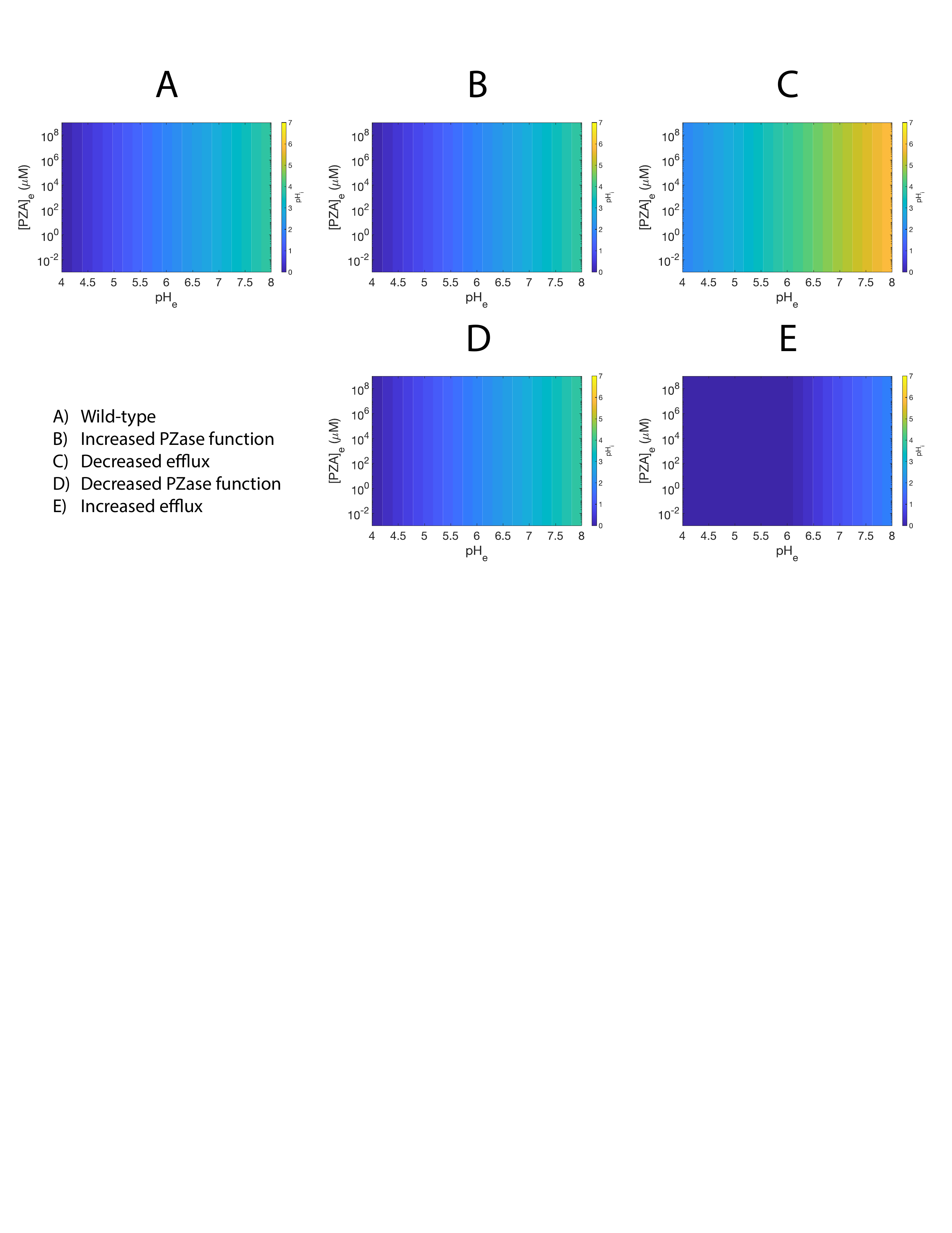

Supplement: S2 Fig — (A) Wild-type MTB. (B) Increased PZase activity. (C) Decreased efflux. (D) Decreased PZase activity. (E) Increased efflux. The color scale is the same in these five panels. (TIFF) [file pone.0309352.s002.tiff]

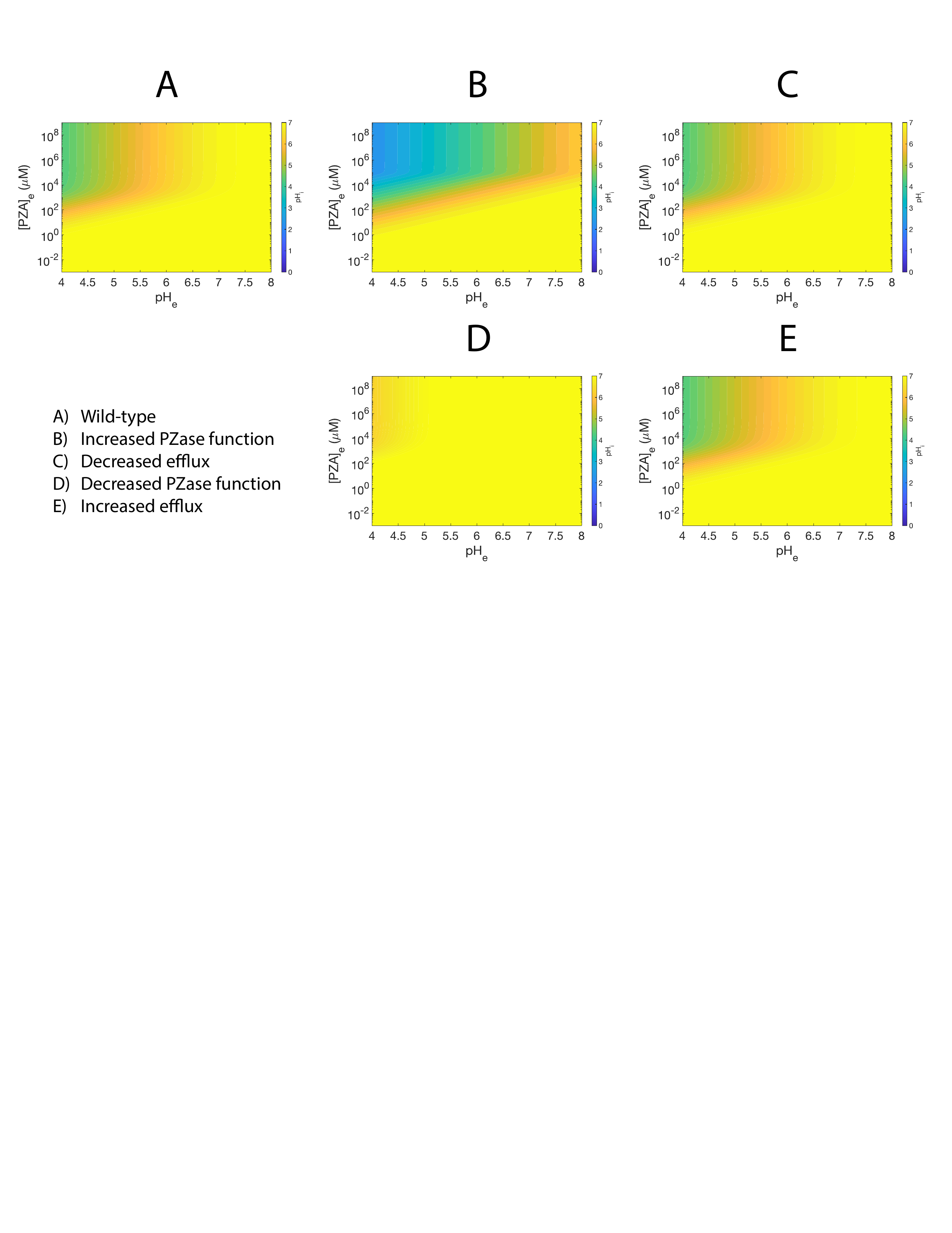

Supplement: S3 Fig — (A) Wild-type MTB. (B) Increased PZase activity. (C) Decreased efflux. (D) Decreased PZase activity. (E) Increased efflux. The color scale is the same in these five panels. (TIFF) [file pone.0309352.s003.tiff]

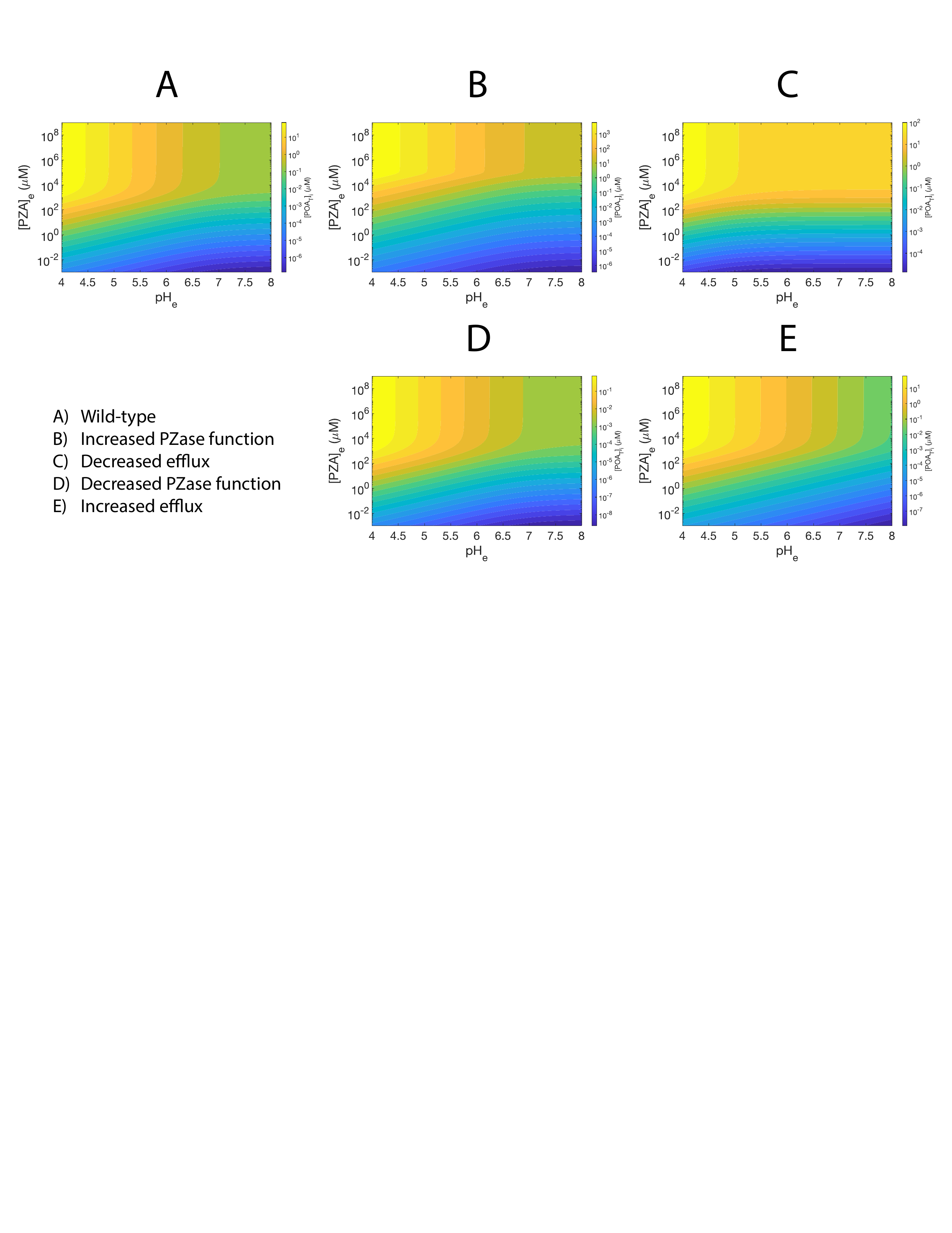

Supplement: S4 Fig — (A) Wild-type MTB. (B) Increased PZase activity. (C) Decreased efflux. (D) Decreased PZase activity. (E) Increased efflux. (TIFF) [file pone.0309352.s004.tiff]

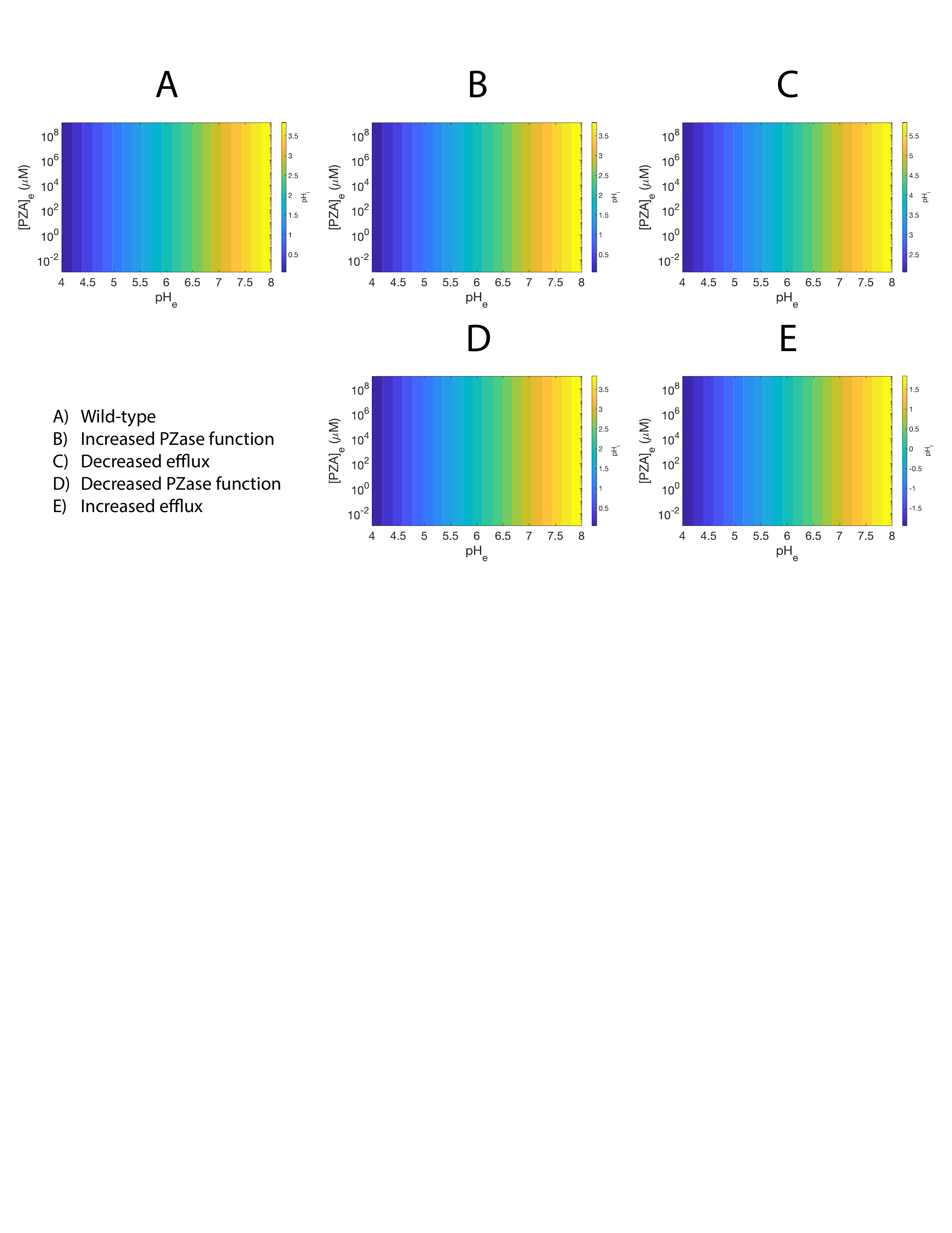

Supplement: S5 Fig — (A) Wild-type MTB. (B) Increased PZase activity. (C) Decreased efflux. (D) Decreased PZase activity. (E) Increased efflux. (TIFF) [file pone.0309352.s005.tiff]

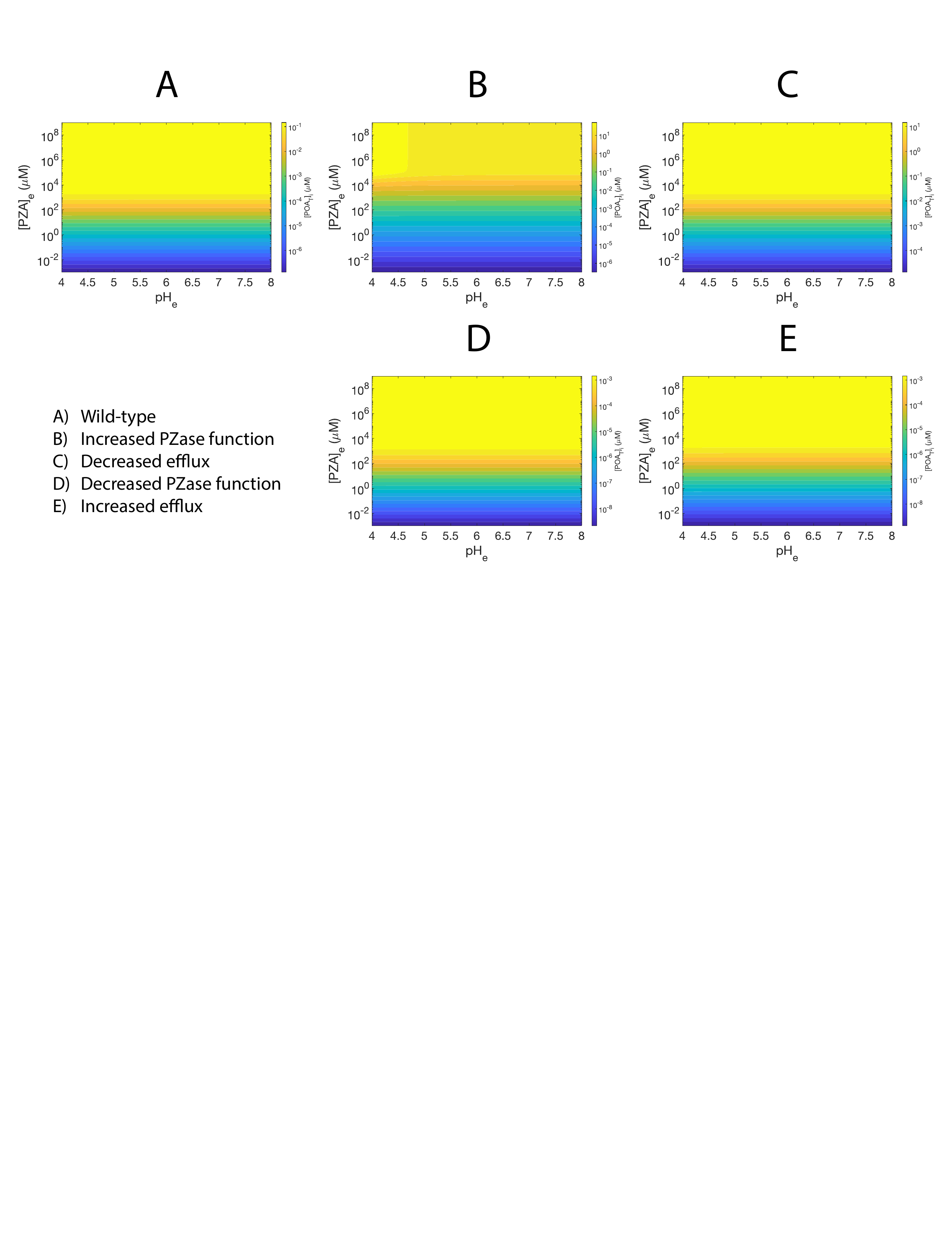

Supplement: S6 Fig — (A) Wild-type MTB. (B) Increased PZase activity. (C) Decreased efflux. (D) Decreased PZase activity. (E) Increased efflux. (TIFF) [file pone.0309352.s006.tiff]

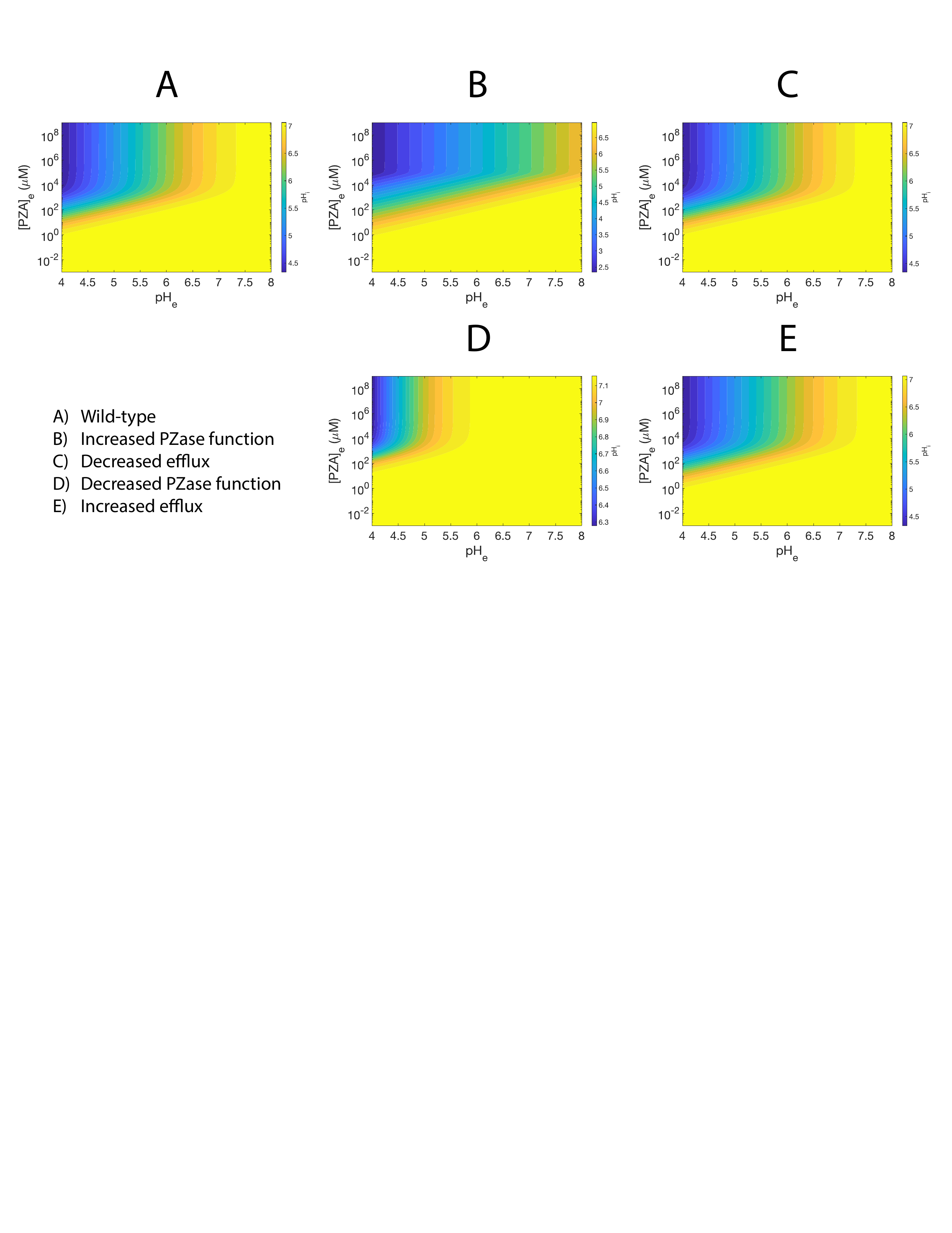

Supplement: S7 Fig — (A) Wild-type MTB. (B) Increased PZase activity. (C) Decreased efflux. (D) Decreased PZase activity. (E) Increased efflux. (TIFF) [file pone.0309352.s007.tiff]
